# Madagascar can build stronger health systems to fight plague and prevent the next epidemic

**DOI:** 10.1371/journal.pntd.0006131

**Published:** 2018-01-04

**Authors:** Matthew H. Bonds, Mohammed A. Ouenzar, Andres Garchitorena, Laura F. Cordier, Meg G. McCarty, Michael L. Rich, Benjamin Andriamihaja, Justin Haruna, Paul E. Farmer

**Affiliations:** 1 Department of Global Health and Social Medicine, Harvard Medical School, Boston, Massachusetts, United States of America; 2 PIVOT Works, Inc., Boston, Massachusetts, United States of America; 3 UMR 224 MIVEGEC, Institut de Recherche pour le Développement, Montpellier, France; 4 Division of Global Health Equity, Brigham and Women’s Hospital, Boston, Massachusetts, United States of America; 5 Madagascar Institute for the Conservation of Tropical Environments, Antananarivo, Madagascar; Baylor College of Medicine, Texas Children's Hospital, UNITED STATES

## Introduction

In August 2017, a 31-year-old man visiting Ankazobe District in the Central Highlands of Madagascar was bitten by a flea that presumably jumped from a cohabitating rat [1]. Within a week, he began to experience malaria-like symptoms as plague-causing bacteria invaded his lymph nodes and then moved to his lungs. En route to the eastern coast, he took a public taxi brousse through the nation’s capital, Antananarivo, and died. The outbreak was officially detected a week later, preceding the infection of more than 2,200 confirmed, probable, and suspected cases as of November 2017, making it one the world’s worst plague epidemics in the past half century [2,3]. Though curable with antibiotics if detected early, more than 200 people have died.

The response of the international community and the national government brought the epidemic significantly under control after some initial delay. Rapid diagnostic tests (RDTs), antibiotics, and protective gear arrived in the capital en masse and were distributed with a host of international actors. Widespread sensitization campaigns were implemented, patients were identified and treated, and thousands of community health workers (CHWs) conducted contact tracing to prevent the spread. However, supply chains and infrastructure throughout Madagascar are weak, and there have been persistent shortages of needed equipment and materials in exposed regions that are traditionally at low risk of plague. The lack of RDTs at many health facilities meant that many cases went unrecognized or were treated empirically at advanced stages, resulting in unchecked transmissions, including to as many as 70 health workers [1]. The risk of a larger epidemic spreading throughout the country this year is now low, but with the seasonal dynamics typically peaking in December and January, vigilance remains critical.

## A familiar pattern in a forgotten place

**Fig 1:**
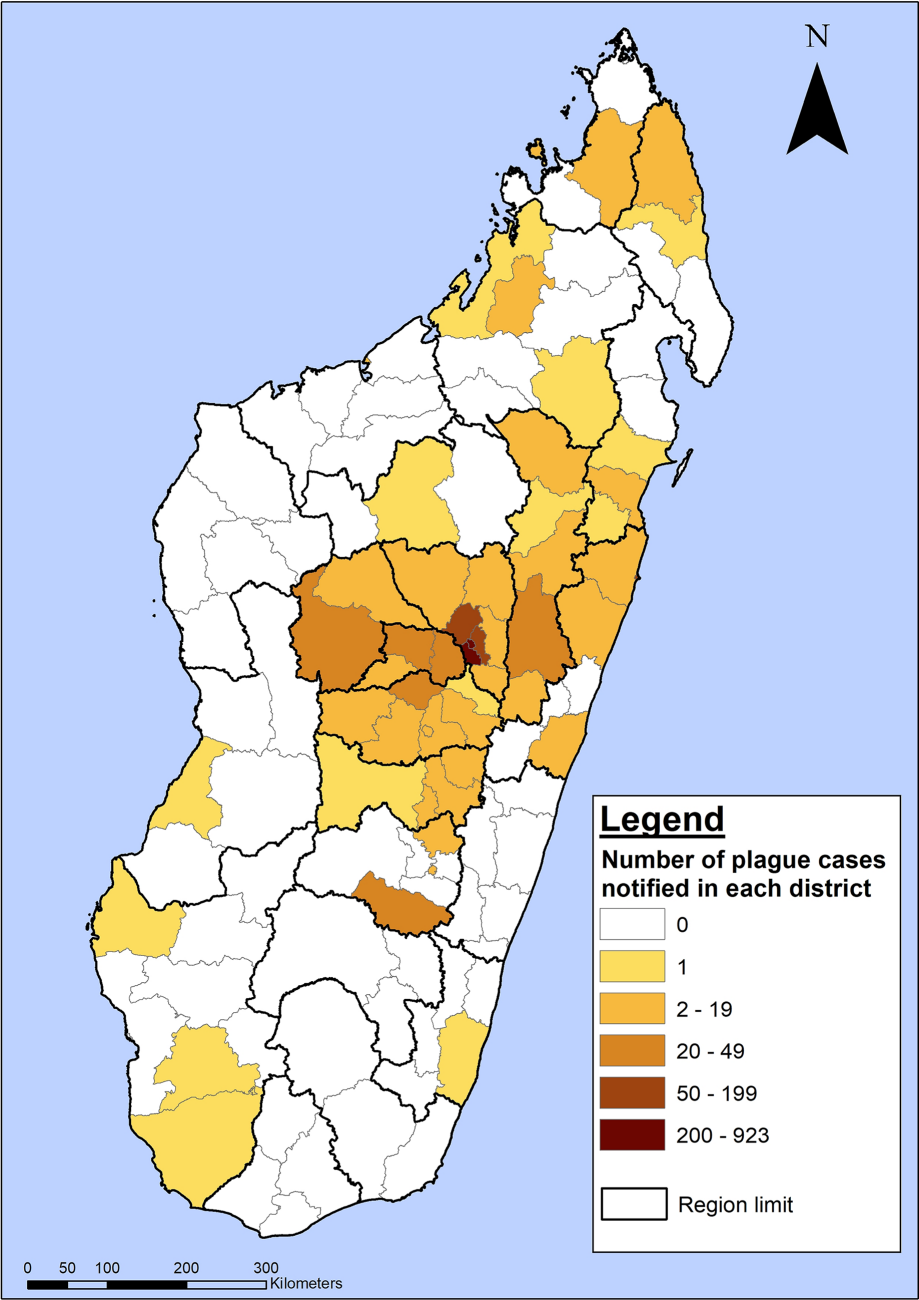
Geographical distribution of plague cases in Madagascar. Over 2,200 cases suspected (e.g., clinical symptoms), probable (e.g., positive RDT), and confirmed (e.g., *Y*. *pestis* isolated via culture) as reported by the World Health Organization as of November 2017 [1]; additional cases country wide were treated as plague based on clinical assessment but not officially reported due to a lack of diagnostic capacity.

Madagascar is well known for its extraordinarily diverse, mostly indigenous species. However, those indigenous species do not include the primary reservoir host of *Yersinia pestis* (the causative agent of plague): the black rat, which invaded the island more than a century ago [4]. Bubonic plague is a zoonotic bacterial infection that causes swelling of the lymph nodes. Complicated cases can lead to infections of the lungs (pneumonic plague), which can then be directly transmitted person-to-person via aerosolized droplets and are fatal without treatment within 48 hours [5]. That there was an outbreak of plague in Madagascar this time of year is no surprise; plague emerges annually, attributed to seasonal environmental conditions that decrease rat populations and drive fleas to feed off of humans directly. Because of the ecological dynamics and persistence of the disease in rats and other hosts, complete eradication is considered nearly impossible [6,7]. But the epidemiological characteristics of plague this year are unique in that it has emerged earlier than is typical, is mostly of the pneumonic form, and struck the capital (Fig 1).

This scenario—a deadly zoonotic infectious disease emerging from a rural community, invading an urban center, and then taking off like wildfire—is an increasing pattern in global epidemics, but it is not inevitable. Root causes in the chronic failures of the health system are well known and similar to those of the Ebola outbreak in West Africa, the Haiti cholera epidemic, and HIV throughout Africa [8]. It is no coincidence that plague has emerged in a country with one of the world’s most underfunded health systems. These failures are also behind the deaths of tens of thousands of children under five to malaria, pneumonia, diarrhea, and other treatable illnesses in Madagascar annually. And as seen before, international responses will likely turn to the next disease outbreak without meaningful commitment to long-term solutions in Madagascar. The chance of a larger plague epidemic in the future will remain unnecessarily high.

The challenge of preventing most causes of illness and death in Madagascar—where treatments are known and affordable—is that seemingly simple solutions require complex delivery systems that align at the point of care. Though there will naturally be calls for greater surveillance and scientific inquiry—including intensifying research and development on plague vaccines [9]—the key to channeling these efforts in order to prevent and control outbreaks is the same for promoting health equity on the whole: stronger health systems.

## A systems solution for Madagascar

**Fig 2:**
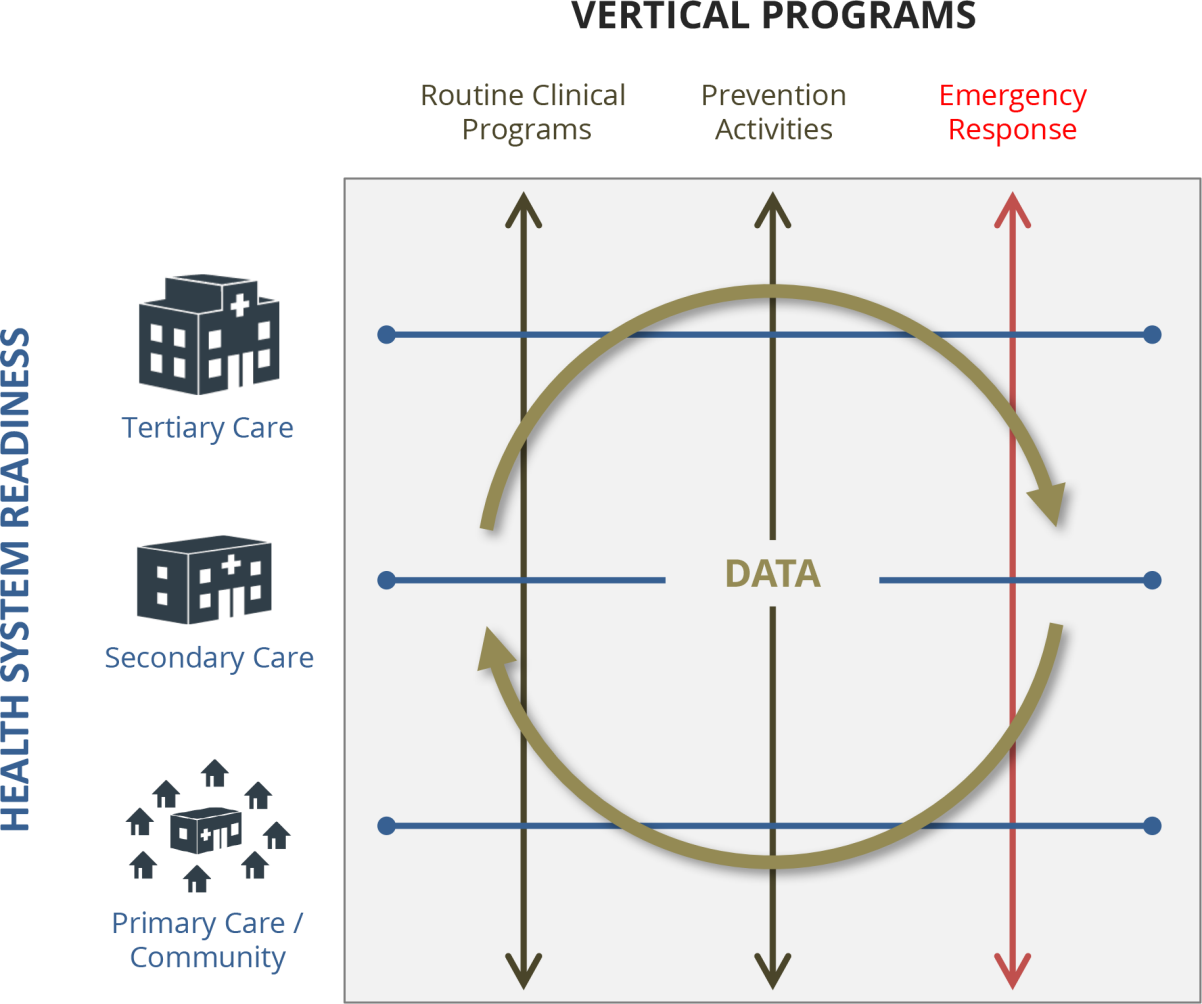
**Strengthened health systems are grounded in three components:** (1) horizontal readiness includes supply chain management, adequate and well-trained staff, medicines, and infrastructure that must be available at all levels of care—primary care through basic health centers and community health programs, secondary hospital care (district hospitals), and tertiary care in national referral and university hospitals; (2) vertically integrated programs include routine clinical programs such as maternal-child health and infectious disease management, prevention activities such as vaccine campaigns, and emergency response to plague and other outbreaks; (3) integrated data from health management information systems and epidemiological surveillance.

Madagascar’s Ministry of Health has strong existing policies to guide its health system but has insufficient resources to implement them. Partly due to a coup in 2009—and the country’s subsequent ineligibility for foreign aid—Madagascar’s annual per capita spending of $14 on health is the lowest in the world [10]. If adequately supported, Madagascar can turn this tragedy into an opportunity to bring its health system into the modern era. This effort to strengthen health systems should be based on three main components (Fig 2), as follows: (1) horizontal “readiness” at all levels of the system, (2) vertically integrated clinical programs, and (3) high-quality data [11].

“Readiness” refers to horizontal capacity—the “staff, stuff, systems, and space”—to support clinical services, including emergency response. There are well-established WHO guidelines (the Service Availability and Readiness Assessment) that can be implemented throughout Madagascar at all levels of the health system—community, primary, secondary, and tertiary care. In cases of plague, the system is “ready” if CHWs are trained to identify and refer sick patients to health centers and if health facilities have the diagnostic capacity, medicines, and trained staff to receive and care for patients.

“Clinical programs” refer to the disease-specific integration of trainings, protocols, drugs, and supplies that must align along the continuum of care for patients to receive appropriate treatment and follow-up for a condition. Importantly, these clinical services can serve as vehicles for strengthening the system overall. Just as 21st-century care for HIV/AIDS played an instrumental role in improving overall health systems in developing countries, the national vertical plague response in Madagascar should strengthen the horizontal capacity to support this and other infectious disease management programs.

There are two key national data systems—health management information systems (HMIS) and epidemiological surveillance—and neither is currently used systematically to improve rapid localized health system response to emergencies. HMIS is based on routine health registry information and is instrumental for national reporting and annual planning. As the national reference laboratory, the Institut Pasteur of Madagascar (IPM) is the leading epidemiological surveillance partner, with sites throughout the country that provide critical diagnostic capacity in the midst of outbreaks; however, they remain fairly independent of routine health care delivery systems. Efforts of electronic Integrated Data Surveillance and Response (eIDSR) and the sentinel network of IPM aim to help improve these efforts, but there remain challenges to integrating these with local health systems to be most effective.

Perhaps the greatest untapped opportunity in Madagascar for preventing and responding to outbreaks are the 40,000 CHWs throughout the country along with the primary care clinical teams that oversee them. Though CHWs are not trained to diagnose or treat plague, they can identify early symptoms; they represent the frontlines of the health system and are the closest knowledge source for the daily well-being of populations. Per national policy, two CHWs are designated to provide services per fokontany (village cluster). And because of Madagascar’s highly dispersed rural populations (most live more than 5 km from the nearest health facility), CHWs have a critical role in identifying the early onset of symptoms and ensuring immediate integration with the health system, including triggering emergency responses. Madagascar has strong policies; however, implementation gaps remain due to a lack of evidence on how to execute national health plans and a paucity of funding. This renders the network of CHWs largely under-equipped and under-supervised, with community-based patient data poorly fed into the national health information systems. Advances in basic mobile technologies can be directly integrated with current community health protocols, with the potential to improve surveillance capabilities and fill in the missing link between national surveillance entities and the frontline health system and communities.

## Conclusion

One of the great myths of global health is that, due to the complexity of social and environmental determinants of disease as well as the complexity of national and local health systems, ensuring that quality health care reaches all people is an intractable challenge. However, the path for doing so is the same path for preventing the next epidemic and is known and achievable: stronger health systems. There are more than 100 independent multilateral and nongovernmental partners in Madagascar. These partners can have a collective impact by aligning with the Ministry of Health, integrating with local communities, and ensuring that existing policies are implemented effectively. To do this, the government and nongovernmental and academic partners must fully cooperate in accordance with the Ministry of Health agenda and make long-term commitments to its implementation. Only with such government-led collaboration will broader attempts to improve surveillance and expand scientific understanding be effective. The result would be a tapestry of greater horizontal readiness, vertically integrated clinical programs, quality data, and a health system that serves all patients. In this way, Madagascar might not only reduce outbreaks of plague but also demonstrate lessons for the global community about health system transformation in an era of global epidemics.
